# A study of structural effects on the focusing and imaging performance of hard X-rays with 20–30 nm zone plates

**DOI:** 10.1107/S1600577524009615

**Published:** 2024-10-28

**Authors:** Xujie Tong, Vishal Dhamgaye, Qiucheng Chen, Qingxin Wu, Biao Deng, Ling Zhang, Oliver Fox, Hongchang Wang, Jun Zhao, Yifang Chen, Zijian Xu, Peng Li, Kawal Sawhney

**Affiliations:** a Nanolithography and Application Research Group, School of Information Science and Technology, Shanghai Key Laboratory of Metasurfaces for Light Manipulation Fudan University Shanghai200433 People’s Republic of China; b Diamond Light Source Ltd Harwell Science and Innovation Campus Didcot OxfordshireOX11 0DE United Kingdom; c Shanghai Synchrotron Radiation Facility, Shanghai Advanced Research Institute Chinese Academy of Sciences Shanghai201210 People’s Republic of China; Tohoku University, Japan

**Keywords:** zone plates, zone structural effects, focusing efficiency, X-ray microscopy, beam-propagation method

## Abstract

Hard X-ray microscopes with 20–30 nm spatial resolution ranges are an advanced tool for the inspection of materials at the nanoscale. However, the limited efficiency of the focusing optics, for example, a Fresnel zone plate (ZP) lens, can significantly reduce the power of a nanoprobe. Despite several reports on ZP lenses that focus hard X-rays with 20 nm resolution – mainly constructed by zone-doubling techniques – a systematic investigation into the limiting factors has not been reported. We report the structural effects on the focusing and imaging efficiency of 20–30 nm-resolution ZPs, employing a modified beam-propagation method. The zone width and the duty cycle (zone width/ring pitch) were optimized to achieve maximum efficiency, and a comparative analysis of the zone materials was conducted. The optimized zone structures were used in the fabrication of Pt-hydrogen silsesquioxane (HSQ) ZPs. The highest focusing efficiency of the Pt-HSQ-ZP with a resolution of 30 nm was 10% at 7 keV and >5% in the range 6–10 keV, whereas the highest efficiency of the Pt-HSQ-ZP with a resolution of 20 nm was realized at 7 keV with an efficiency of 7.6%. Optical characterization conducted at X-ray beamlines demonstrated significant enhancement of the focusing and imaging efficiency in a broader range of hard X-rays from 5 keV to 10 keV, demonstrating the potential application in hard X-ray focusing and imaging.

## Abstract 2

This work reports the structural effects on the focusing and imaging efficiency of 20–30 nm-resolution zone plates at Diamond Light Source and Shanghai Synchrotron Radiation Facility. The zone width and the duty cycle were optimized using a modified beam-propagation method for peak efficiency, and the effect of residual resist was also explored using simulation and experimental measurements.

## 1. Introduction

Hard X-ray nanoprobes with sub-30 nm focusing and imaging resolution, as well as high efficiency, are in high demand owing to their potential for broad applications in material science, biomaterials, geology and environmental science (Santos *et al.*, 2021 ▸; Fan *et al.*, 2018 ▸) as well as the measurement of biological samples (Yamamoto *et al.*, 2017 ▸; Dehlinger *et al.*, 2020 ▸; Ferreira Sanchez *et al.*, 2021 ▸; Chen *et al.*, 2021 ▸; Le *et al.*, 2019 ▸). Fresnel zone plates (FZPs) with 20 nm resolution are the key components in many hard X-ray nanoprobes and have been fabricated from various materials including gold (Gorelick *et al.*, 2011 ▸; Tong *et al.*, 2022 ▸), tungsten (Tiwari *et al.*, 2017 ▸), quartz (Moldovan *et al.*, 2018 ▸) and silicon (Simons *et al.*, 2016 ▸). A variety of techniques such as the zone-doubling process (Mohacsi *et al.*, 2017 ▸), multilayer depositions (Eberl *et al.*, 2014 ▸; Osterhoff *et al.*, 2017 ▸), electron-beam lithography (EBL) (Uhlén *et al.*, 2011 ▸, 2014 ▸; Moldovan *et al.*, 2018 ▸) and metal-assisted chemical etching (Li *et al.*, 2020 ▸, 2017 ▸) have been reported previously in the fabrication of high-resolution FZPs. Despite achieving a 20 nm resolution, the focusing and imaging efficiency of FZPs remains suboptimal, which restricts the capabilities of high-resolution X-ray nanoprobes. This inefficiency is primarily due to a limited understanding of how the nanostructures and materials used in the ZPs influence their performance. When the FZP spatial resolution drops to sub-30 nm, the zone structure and material become crucial in determining X-ray propagation and the ZP focusing/imaging properties, offering opportunities to optimize the nano-structure and material of the device to enhance performance. In our earlier work, compound ZPs (CZPs) made from HfO_2_-hydrogen silsesquioxane (HSQ) for the outer zones were first developed (Tong *et al.*, 2023 ▸) using the beam-propagation method (BPM) simulation with soft X-rays. A zone-doubling method (Jefimovs *et al.*, 2007 ▸) was applied to form the 15 nm outermost zone width in HfO_2_ and an efficiency as high as 7.4% was achieved for soft X-ray focusing.

In this study, we present Pt-HSQ ZPs with 20 nm and 30 nm outermost zone widths for hard X-ray focusing and imaging. A Pt film was coated by atomic layer deposition (ALD) onto the side-walls of pre-generated HSQ templates to form Pt-HSQ ZPs. To enhance efficiency, both the duty cycle and the outermost zone width along with the thickness of the Pt film deposited by ALD were optimized. A systematic study of the relationship between the zone structure/material and the focusing/imaging performance was undertaken, with the objective of identifying the optimal structural parameters for achieving maximum efficiency. Utilizing these optimized parameters, hard X-ray ZPs with 20 nm and 30 nm resolution were fabricated by employing state-of-the-art EBL methods. Optical characterization of the focusing/imaging capability was performed using transmission X-ray microscopy (TXM) and ptychographic coherent diffraction imaging (CDI). The fabricated compound Pt-HSQ-ZPs demonstrated encouraging focusing efficiency at hard X-ray energies for practical applications in synchrotron nanoprobe beamlines.

## 2. Optimal structural design of zone-doubled zone plates

**Figure 1:**
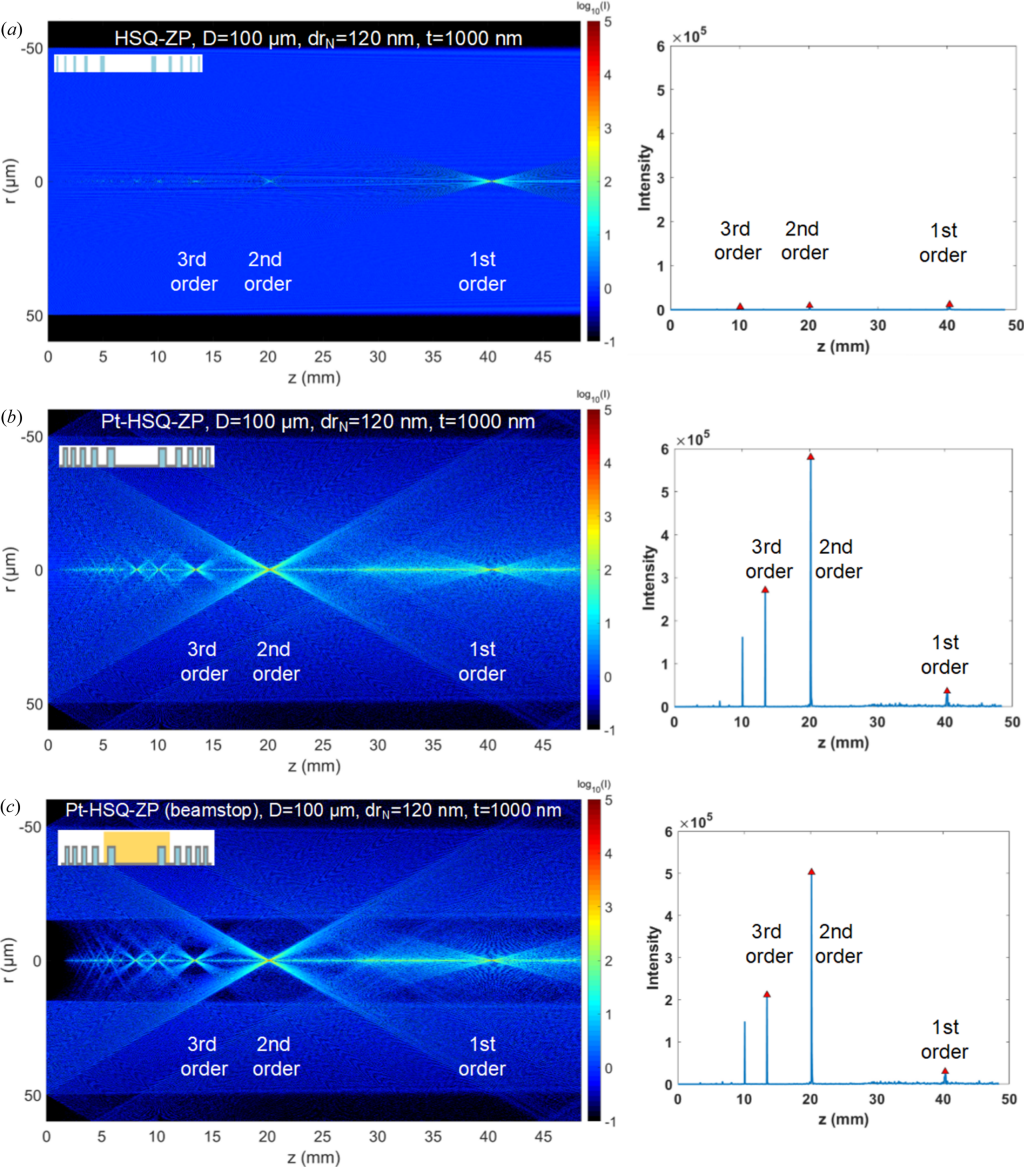
Calculated results of wavefield propagation of ZPs using a modified BPM (Tong *et al.*, 2022 ▸) for (*a*) the ZP template before ALD (HSQ-ZP), (*b*) the ZP after ALD of Pt (Pt-HSQ-ZP) and (*c*) the ZP integrated with a beamstop (30 µm diameter). The X-ray energy was 8 keV and the ZP resolution was 30 nm. The plots on the right show the light field intensity distribution extracted along the central optical axis.

In order to analyze the impact of structural effects of the zone-doubled ZP on efficiency, the intensity distributions through the HSQ-ZP and Pt-HSQ-ZP with and without a beamstop (30 µm diameter) were calculated by the modified BPM and are shown in Fig. 1 ▸. The modified BPM is based on a radial 2D model of the compound ZP using the circular symmetry of the Fresnel ZP. The HSQ mold and ALD material were taken into account according to their index and structure functions; the detailed calculation method is described elsewhere (Tong *et al.*, 2022 ▸, 2023 ▸). For a 30 nm spatially resolved ZP, a 100 µm diameter, 1000 nm height (*t*) and 8 keV X-ray energy were used in the calculation. Fig. 1 ▸(*a*) shows the wavefield through the HSQ-ZP lens before the Pt film deposition. The HSQ-ZP with a height of 1000 nm has almost no focusing ability for hard X-rays. The 2D far-field intensity distribution map shows that the hard X-ray modulation ability of the HSQ-ZP is weak due to low-*Z* materials which have lower electron densities that reduce the phase shift and diffraction efficiency. Therefore, the focal spot intensity of the first order of the HSQ-ZP is negligible and a large amount of light passes through the lens, causing unwanted background illumination on the focal plane. The far-field light intensity distribution of the Pt-HSQ-ZP lens after the deposition of 30 nm Pt on the side-walls of the HSQ-ZP is shown in Fig. 1 ▸(*b*). The Pt film acts as an effective ZP with doubled periods. The focus spot intensity for the Pt-HSQ-ZP is significantly higher than that of the HSQ-ZP with a theoretical efficiency of 11.9%. The light field intensity distribution with a beamstop to block the zeroth-order light is shown in Fig. 1 ▸(*c*), causing a loss of the intensity transmitted through the lens by 10% but improving the signal-to-noise ratio.

**Figure 2:**
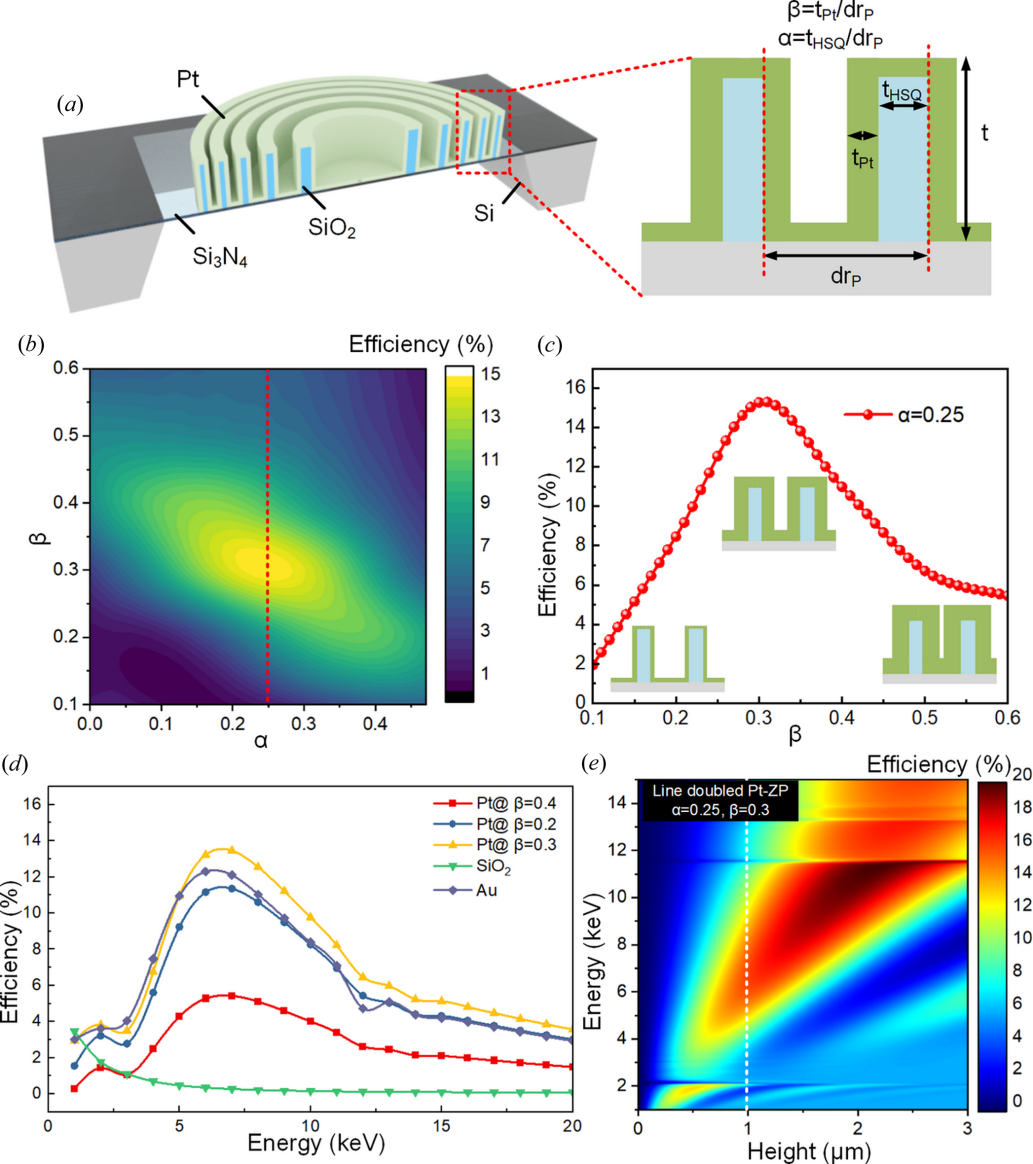
Calculation results for the efficiencies of the Pt-HSQ-ZP. (*a*) Illustration of the zone-doubled ZP structure. (*b*) 2D efficiency map of the zone-doubled ZP with varying ALD ratio and duty-cycle ratio calculated using the BPM. The diameter of the ZP was 100 µm with an outermost zone width of 30 nm and a height of 1 µm. The X-ray energy was fixed at 7.2 keV. (*c*) Plot of efficiency changes with the ALD ratio from (*b*) when the duty-cycle ratio is fixed at α = 0.25. (*d*) Focusing efficiency calculated using the BPM for the HSQ-ZP coated with a high refractive index film of different materials by ALD. The diameter of ZPs was 100 µm and the height was 1 µm. (*e*) Theoretical focusing efficiency of the Pt zone-doubled ZP as a function of X-ray energy and lens height calculated using the BPM.

Fig. 2 ▸(*a*) is an illustration of the Pt-HSQ-ZP, where the outermost ring-pitch of the lithographically patterned HSQ-ZP is *dr*_P_ and the outermost ring-width is *t*_HSQ_. The influence of the duty-cycle ratio, α = *t*_HSQ_/*dr*_P_, on the focusing performance was studied. The thickness of the Pt layer (*t*_Pt_) for the Pt zone gives rise to another duty-cycle ratio where β = *t*_Pt_/*dr*_P_. The BPM results indicate that values of α = 0.25 and β = 0.25 should provide the ideal zone-doubled ZP.

For 30 nm resolution, the outermost ring pitch (*dr*_P_) of the HSQ-ZP was fixed at 120 nm and the zone height, *t*, was 1 µm. The outermost ring-width of the Pt-HSQ-ZP was optimized. Fig. 2 ▸(*b*) shows the calculated efficiencies varying with the duty-cycle ratio of α and β. The peak efficiency occurs at a duty-cycle ratio of α = 0.25, which is a quarter of the outermost ring-pitch of the HSQ-ZP. However, for the Pt-HSQ-ZP, the maximum efficiency was reached when the duty-cycle ratio β ≃ 0.3, as seen in Fig. 2 ▸(*c*), which is slightly larger than the ideal value (0.25), indicating that slight over-deposition of the Pt layer by 20% is beneficial for the focusing efficiency of the Pt-HSQ-ZP. When β = 0.25, all zones except the outermost ring are under-deposited, causing a reduction in the overall focusing efficiency.

The effect of the deposited material on the focusing performance was also addressed using the BPM (Tong *et al.*, 2022 ▸). Fig. 2 ▸(*d*) shows a comparison of the efficiency of the ZP with various materials over a broad energy range for a fixed zone height of 1 µm. Fig. 2 ▸(*e*) shows a map of the focusing efficiency of the Pt-HSQ-ZP as a function of X-ray energy and ZP height under the optimal duty-cycle ratios (α = 0.25, β = 0.3). The focusing efficiencies reach a maximum around 7 keV for the zone materials of Pt and Au. With Pt as the zone material and duty-cycle ratios of β = 0.2, 0.3 and 0.4, a 35 nm-thick Pt layer gives rise to the highest efficiency. SiO_2_ is almost transparent to hard X-rays, resulting in low efficiency, indicating that the HSQ-ZP acts as a scaffold only. The focusing efficiencies of Pt-HSQ-ZPs with three different Pt thicknesses are also shown in Fig. 2 ▸(*d*), and when β deviates from the ideal value of 0.3, the focusing efficiency decreases as expected. Increasing the Pt thickness by 20% improves the focusing efficiency in all zones except the outermost one. Therefore, the optimal duty-cycle β (*t*_Pt_/*dr*_P_) ranges from 0.25 to 0.35 for the peak focusing efficiency of the Pt-HSQ-ZP at 7 keV.

## 3. Fabrication and optical characterizations of optimized zone plates

**Figure 3:**
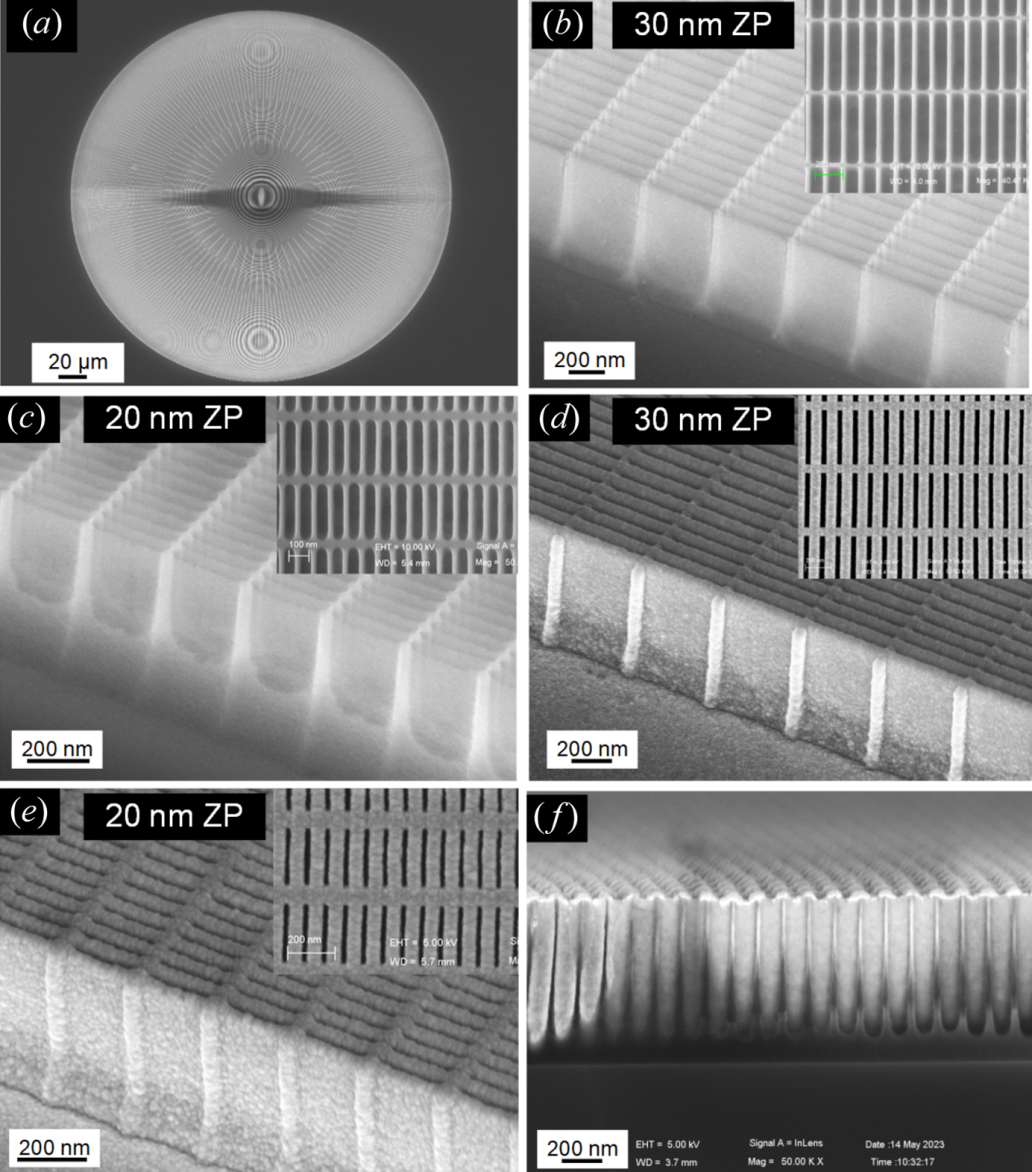
SEM micrographs for the fabricated ZPs. (*a*) Overview of the HSQ-ZP template with a diameter of 300 µm, *t* = 1000 nm. (*b*, *c*) SEM images at a tilt of 45° of the HSQ-ZP. (*d*, *e*) SEM images at a tilt of 45° of the HSQ-ZP after ALD of Pt. The inserts show the top view of the outer zones using the same scale bar as the tilted images. (*f*) Cross-sectional SEM view of the Pt-HSQ-ZP on an Si wafer substrate, observed with a tilt angle of 5°.

The Pt-HSQ-ZPs, designed with outermost zone widths of 20 nm and 30 nm, were fabricated using EBL followed by ALD. A 1000 nm-thick SiO_*x*_ based HSQ layer was spin-coated onto a 300 nm-thick Si_3_N_4_ membrane. EBL exposure was completed in a JBX-6300 FS beam writer at 100 kV with a 7 nm beam diameter, hot-developing (Chen, 2015 ▸) in tetra­methyl­ammonium hydroxide (TMAH):H_2_O (1:3) was then applied. Finally, the outer zones were formed by ALD of Pt on the side-walls of the HSQ zones. The scanning electron microscope (SEM) micrographs in Fig. 3 ▸(*a*) show the overview of the HSQ ZP. Figs. 3 ▸(*b*) and 3 ▸(*c*) show the smooth surface and the ideal duty cycle of an HSQ-ZP before ALD with outermost widths of 30 nm and 20 nm, respectively. For the 30 nm-resolution ZP, the outer zones are rectangular in shape, where the outermost ring-width is 30 nm with a pitch of 120 nm, giving rise to an aspect ratio of 33:1 (α = 0.25, β = 0.25) which is within the process error range. For the 20 nm-resolution ZP, a residual resist is observed between the HSQ structures, reducing the effective height and lowering the focusing efficiency. Fig. 3 ▸(*d*) shows the structure after 30 nm Pt has been deposited on the side-walls of the HSQ-ZP. The remaining gap at the outermost ring is 30 nm, forming the period doubling as reported (Vila-Comamala *et al.*, 2012 ▸). The 20 nm-resolution Pt-HSQ-ZP is shown in Fig. 3 ▸(*e*). The outermost gap obtained was 18 nm (α = 0.25, β = 0.28), acceptable for the high performance of the Pt-HSQ-ZP at 7.2 keV. It can be observed in the SEM images that the Pt film quality on the side-walls of the 20 nm ZP is good, with only fine particles caught on the surface. Fig. 3 ▸(*f*) shows a cross-sectional view of the fabricated Pt-HSQ-ZP on an Si wafer at a 5° tilt, with uniform Pt deposition on both sides of the HSQ structures.

The imaging resolution of the fabricated zone-doubled ZPs was evaluated at the optical test bench at the Shanghai Synchrotron Radiation Facility (SSRF), using TXM with hard X-rays on the BL18B beamline. The setup utilized a monocapillary condenser to focus an X-ray beam with an energy of 7.2 keV onto a Siemens star test pattern. A pinhole was positioned between the condenser and the Siemens star to filter out any direct light not originating from the capillary, ensuring a clean beam. The Siemens star test samples were fabricated in-house with a resolution of 20 nm and 30 nm and had a structure height of 1.5 µm. The zone-doubled FZP then magnified and imaged the test pattern onto a water-cooled sCMOS detector (Andor Zyla 4.2 Plus).

**Figure 4:**
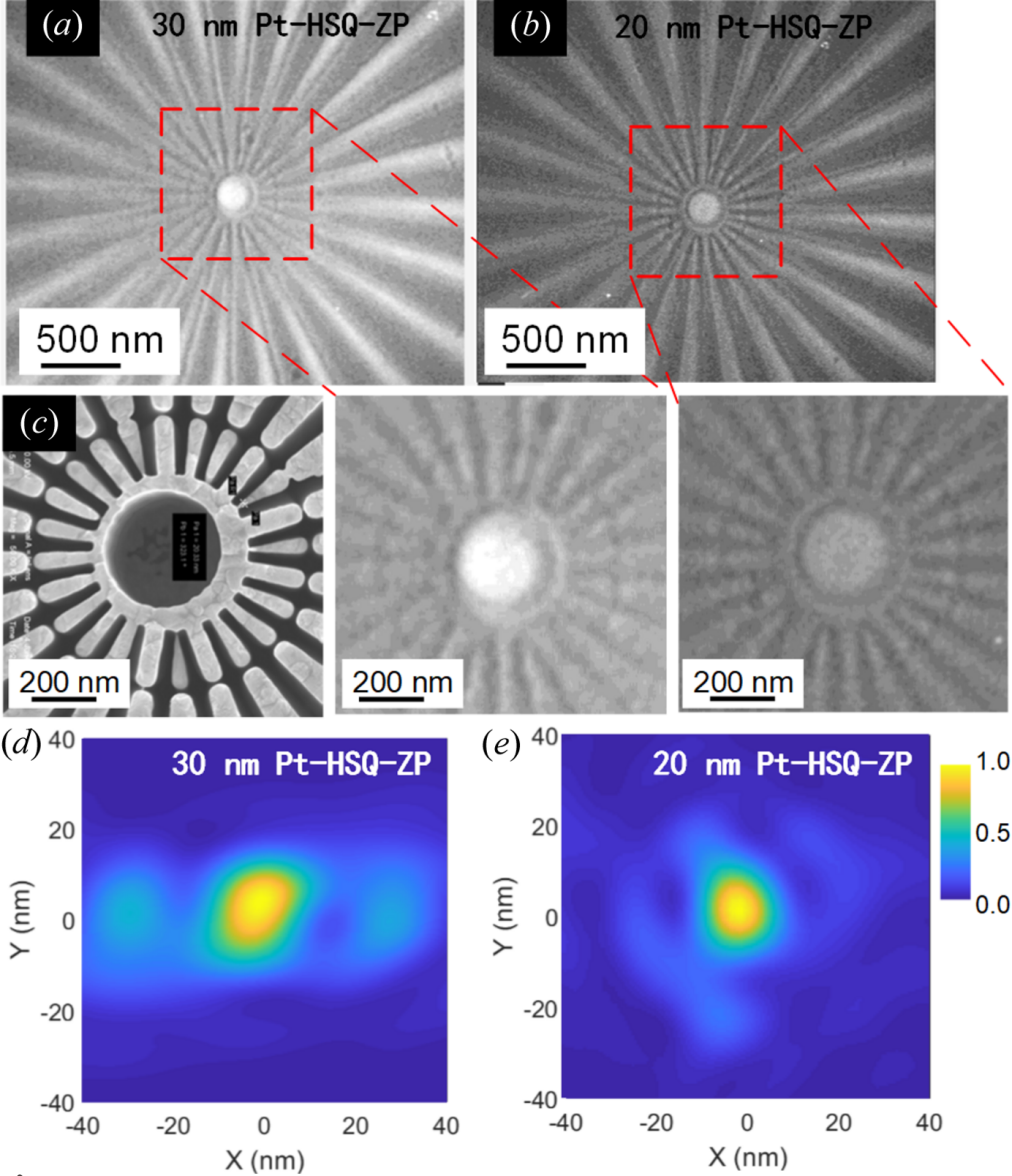
Focusing and imaging quality of the Pt-HSQ-ZPs measured using TXM. TXM images of a Siemens star test sample using the Pt-HSQ-ZPs with resolutions of (*a*) 30 nm and (*b*) 20 nm. The energy was 7.2 keV and the dwell time was 10 s. (*c*) SEM image of the Siemens star with a minimum resolution of 20 nm. Focusing performance characterization using X-ray ptychographic CDI and 2D intensity distribution for (*d*) 30 nm and (*e*) 20 nm Pt-HSQ-ZPs.

Fig. 4 ▸ presents the results of TXM imaging of the Siemens star using the Pt-HSQ-ZPs. Fig. 4 ▸(*a*) illustrates the imaging results obtained with 30 nm-resolution Pt-HSQ-ZP. The TXM image was captured using a dwell time of 10 s and a pixel size of 11 nm (Zhang *et al.*, 2023 ▸). The image shows good contrast; however, the 20 nm features in the central region of the Siemens star are barely resolved. For comparison, the same imaging procedure was performed on the same Siemens star using the Pt-HSQ-ZP with a 20 nm resolution, as shown in Fig. 4 ▸(*b*). Comparing imaging results, the 20 nm-resolution Pt-HSQ-ZP demonstrates superior performance, providing more detailed features in the central area where the imaging resolution is ∼24 nm. However, under the same incident beam flux, the image contrast is lower compared with the 30 nm Pt-HSQ-ZP. This reduction in contrast is attributed to decreased efficiency caused by the residual resist present in the HSQ trenches.

The focusing properties of the optimized Pt-HSQ-ZPs were characterized at the B16 Test Beamline of Diamond Light Source (DLS) (Sawhney *et al.*, 2010 ▸). A ptychographic CDI technique was employed to obtain complete spatial information of the complex probe function. A Siemens star test object was positioned ∼2–3 mm downstream of the ZP focal plane on Attocube piezoelectric translational stages. A third-generation Merlin Medipix single-photon-counting detector with a pixel size of 55 µm × 55 µm (512 × 512 pixels) was positioned ∼2140 mm downstream of the test object. Ptychography scans were conducted with an incident X-ray energy of 7.2 keV, stepping the test object through the beam from the ZP and capturing diffraction patterns at each sample position. For the 30 nm-resolution ZP, the ptychography scan was performed over an area of 5 µm × 5 µm with a step size of 300 nm along an Archimedean spiral path consisting of 274 points and the linear overlap ratio was approximately 85%. For the 20 nm-resolution ZP, a similar ptychography scan was performed and the linear overlap was ∼92.5%. The complex probe and object functions were reconstructed accurately using a multi-mode extended ptychographic iterative algorithm (Maiden & Rodenburg, 2009 ▸; Thibault & Menzel, 2013 ▸) with 500 iterations. Then, the reconstructed probe function was back-propagated to the focal plane of the ZP. The 2D intensity distributions in the focal plane of both Pt-HSQ-ZPs are shown in Figs. 4 ▸(*d*) and 4 ▸(*e*). The measured (theoretical) full widths at half-maxima of the reconstructed focal spot were found to be 35 (27) nm and 22 (18) nm for the Pt-HSQ-ZP with resolutions of 30 nm and 20 nm, respectively. For a 30 nm-resolution ZP, side lobes in the *x* direction were observed, indicating a slightly distorted wavefront. For the 20 nm-resolution ZP, the reconstructed wavefront is in good agreement with the theoretical focal pattern and the background noise is effectively suppressed.

Focusing efficiency tests were also conducted at the B16 Test Beamline. The intensity concentrated into the first-order focus of the ZP was measured. To determine the diffraction efficiency of an FZP, two intensity measurements were performed: (1) the focal intensity of the first order was obtained by measuring the intensity with the FZP, beamstop and OSA in the beam path; and (2) the incident intensity was measured by removing the FZP. Then, the diffraction intensity was calculated as the ratio of these two measured intensities after subtracting the background signal (Tong *et al.*, 2022 ▸). The initial measurements were carried out at a photon energy of 7.2 keV. The efficiency trends of the ZPs with the two resolutions were similar to the theoretical calculations, reaching the maximum value at 7 keV. The maximum focusing efficiency of the Pt-HSQ-ZP with 30 nm resolution is about 10%. For photon energies in the range 6–10 keV, it maintained an efficiency above 5%. The maximum efficiency of Pt-HSQ-ZP with 20 nm resolution is 7.6% with an average focusing efficiency of 5.8% between 6 keV and 10 keV, which is higher than most reported values (Moldovan *et al.*, 2018 ▸; Li *et al.*, 2020 ▸; Sanli *et al.*, 2018 ▸).

**Figure 5:**
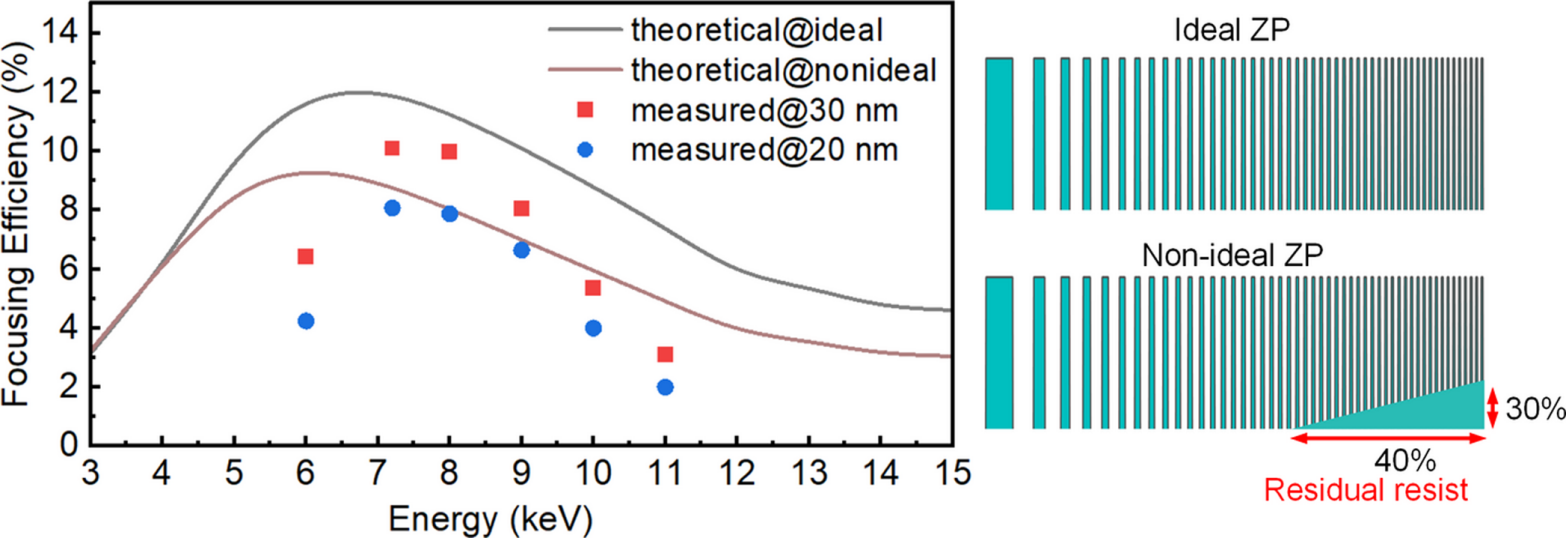
Comparison of focusing efficiencies between Pt-HSQ-ZPs without (ideal) and with (non-ideal) residual resist in the HSQ trenches, highlighting the effect on the lens efficiency. Both the 20 nm- and the 30 nm-resolution ZPs are included. The lens diameter was 300 µm and the zone height was 1000 nm.

As shown in Fig. 3 ▸(*f*), a residual photoresist was observed on the periphery of the 20 nm-resolution Pt-HSQ-ZP, reducing the effective zone height. To address the influence of the residual resist on the performance of the 20 nm-resolution Pt-HSQ-ZP and to study the trade-off between the resolution and efficiency, we modeled a non-ideal ZP using the BPM based on the quasi-discrete Hankel transform (Tong *et al.*, 2022 ▸). We reconstructed the radial function of the non-ideal composite lens with structural deviations and refractive index information, as shown in Fig. 5 ▸. The zone-doubling structure at the bottom was replaced by HSQ material. BPM calculations indicated that the focusing efficiency of the non-ideal Pt-HSQ-ZP with residual resist at 7.2 keV was lower than that of the ideal ZP. The loss of effective phase shift in the outer area explained the lower efficiency of the 20 nm-resolution ZP compared with the 30 nm one. Therefore, it is crucial to maintain distinct trenches in patterned HSQ for the fabrication of high-resolution ZPs. Additionally, outside of the optimal operating energy range 7–9 keV, a greater deviation between the measured efficiency and the ideal value was observed due to the large background noise.

## 4. Conclusions and outlook

We have investigated the influence of the duty cycle and the outermost zone width determined by the thickness of the Pt film deposited by ALD on the focusing efficiency of the 20–30 nm Pt-HSQ-ZPs, investigated using our modified BPM. The optimal structure ratio of the Pt zone width to the zone pitch was found in the region of 0.25–0.35, resulting in the maximum efficiency at 7.2 keV for the particular ZPs examined. Based on the optimized structural parameters from the BPM calculation, we successfully fabricated 20 nm- and 30 nm-resolution Pt-HSQ-ZPs with a height of 1 µm using the zone-doubling technique. Optical characterizations were performed using TXM at the BL18B Beamline Station of SSRF resulting in high-quality images of the test object with a spatial resolution down to 24 nm.

Efficiency measurements and focal spot characterization using X-ray ptychographic CDI were conducted at the B16 Test Beamline of DLS. The Pt-HSQ-ZP with 30 nm resolution exhibited a maximal focusing efficiency of 10% at 7 keV, and maintained an efficiency above 5% in the photon energy range 6–10 keV. Conversely, the 20 nm-resolution Pt-HSQ-ZP achieved a peak efficiency of 7.6% at 7 keV, which is understood to be caused by the residual resist remaining in the HSQ trenches after fabrication. With our background in high-resolution EBL, we are confident that further efficiency enhancements in high-resolution focusing and imaging are achievable, addressing the pressing demands in the various applications on synchrotron beamlines.

## Data Availability

Data underlying the results presented in this paper are not publicly available at this time but may be obtained from the authors upon reasonable request.
